# Exposure to ambient air pollution and cognitive function: an analysis of the English Longitudinal Study of Ageing cohort

**DOI:** 10.1186/s12940-024-01075-1

**Published:** 2024-04-05

**Authors:** Dylan Wood, Dimitris Evangelopoulos, Sean Beevers, Nutthida Kitwiroon, Panayotes Demakakos, Klea Katsouyanni

**Affiliations:** 1 Environmental Research Group, School of Public Health, Imperial College, Sir Michael Uren Hub, 86 Wood Ln, London, W12 0BZ UK; 2 MRC Centre for Environment and Health, Environmental Research Group, Imperial College, London, W12 0BZ UK; 3 NIHR HPRU in Environmental Exposures and Health, Imperial College, London, UK; 4 Department of Epidemiology and Public Health, University College London (UCL), London, UK; 5 Department of Hygiene, Epidemiology and Medical Statistics, School of Medicine, National and Kapodistrian University of Athens, Athens, Greece

**Keywords:** Cognitive function, Air pollution, Environmental risk factors

## Abstract

**Background:**

An increasing number of studies suggest adverse effects of exposure to ambient air pollution on cognitive function, but the evidence is still limited. We investigated the associations between long-term exposure to air pollutants and cognitive function in the English Longitudinal Study of Ageing (ELSA) cohort of older adults.

**Methods:**

Our sample included 8,883 individuals from ELSA, based on a nationally representative study of people aged ≥ 50 years, followed-up from 2002 until 2017. Exposure to air pollutants was modelled by the CMAQ-urban dispersion model and assigned to the participants’ residential postcodes. Cognitive test scores of memory and executive function were collected biennially. The associations between these cognitive measures and exposure to ambient concentrations of NO_2_, PM_10_, PM_2.5_ and ozone were investigated using mixed-effects models adjusted for time-varying age, physical activity and smoking status, as well as baseline gender and level of education.

**Results:**

Increasing long-term exposure per interquartile range (IQR) of NO_2_ (IQR: 13.05 μg/m^3^), PM_10_ (IQR: 3.35 μg/m^3^) and PM_2.5_ (IQR: 2.7 μg/m^3^) were associated with decreases in test scores of composite memory by -0.10 (95% confidence interval [CI]: -0.14, -0.07), -0.02 [-0.04, -0.01] and -0.08 [-0.11, -0.05], respectively. The same increases in NO_2_, PM_10_ and PM_2.5_ were associated with decreases in executive function score of -0.31 [-0.38, -0.23], -0.05 [-0.08, -0.02] and -0.16 [-0.22, -0.10], respectively. The association with ozone was inverse across both tests. Similar results were reported for the London-dwelling sub-sample of participants.

**Conclusions:**

The present study was based on a long follow-up with several repeated measurements per cohort participant and long-term air pollution exposure assessment at a fine spatial scale. Increasing long-term exposure to NO_2_, PM_10_ and PM_2.5_ was associated with a decrease in cognitive function in older adults in England. This evidence can inform policies related to modifiable environmental exposures linked to cognitive decline.

**Supplementary Information:**

The online version contains supplementary material available at 10.1186/s12940-024-01075-1.

## Background

Cognitive decline and dementia incidence are growing problems in ageing societies [27]. Twenty-one environmental and other modifiable risk factors which may be prioritised for prevention policies have been identified [19] and among those is higher air pollution exposure. Indeed, there is increasing evidence of adverse associations between exposure to ambient air pollution and cognitive outcomes in adult populations [7, 21, 28, 39].

Specifically, an accumulating body of evidence indicates adverse associations between long-term exposure to pollutants (especially fine particulate matter) and global cognition, domain-specific cognitive function or cognitive decline. In terms of reviews, Clifford et al. [7] concluded that the evidence is consistent for an association between exposure to traffic-related pollutants and cognitive decline in the elderly, with this supported by possible toxicological mechanisms. In a critical review of the epidemiological literature on dementia and cognitive decline, Delgado-Saborit et al. [10] reported consistent adverse associations between long-term exposure to ambient air pollution and cognitive function. However, the authors noted greater heterogeneity in associations reported between exposure to ambient air pollution and cognitive function in sub-domains including memory and executive function. These conclusions were echoed in a recent review of relevant epidemiological studies by the Committee on the Medical Effects of Air Pollutants (COMEAP), within which the authors of Delgado-Saborit et al. [10] contributed [8]. Weuve et al. [39] conducted a comprehensive review of studies investigating air pollution exposure and dementia (including dementia-related outcomes), as well as studies on global and domain-specific cognitive function and decline. The review concluded that the evidence evaluating the role of particulate matter ≤ 2.5 µm in diameter (PM_2.5_) exposure in cognitive decline tends to indicate adverse effects but considered the results collectively inconclusive. Thompson et al. [36] conducted a meta-analysis of 14 studies in adult populations and found increasing nitrogen dioxide (NO_2_) and PM_2.5_ to be associated with lower performance in global cognitive test scores, as well as increasing PM_2.5_ to be associated with decreasing performance in executive function tasks.

Two cohort studies have assessed the effects of exposure to air pollution on cognitive function in UK adults to date [9, 37]. Tonne et al. [37] analysed cognitive change over five years in 2,867 participants of the Whitehall II cohort and reported no associations between increasing particulate matter ≤ 10 µm in diameter (PM_10_) and PM_2.5_ exposures and decline in test scores, although restricting the sample to those that remained in London throughout the course of follow-up did suggest a reduction in memory test score per 1.8 and 1.1 µg/m^3^ increase in PM_10_ and PM_2.5_ exposure four years preceding the second assessment, respectively. A cross-sectional analysis did observe small reductions in performance on a test of reasoning ability in a larger sample of 3,414 individuals. Cullen et al. [9] reported some associations between pollutants and test results targeted at specific cognitive domains in a cross-sectional analysis in the UK Biobank cohort (*n* = 86,759), whilst finding no associations between pollutant exposure and decline in test scores of memory and executive function through a sub-sample of individuals that provided a repeated measure (*n* = 2,913).

Weuve et al. [39] note methodological challenges concerning the short follow-up periods and limited number of follow-ups in relevant cohort studies of cognition in relation to air pollution exposure, as well as the often relatively small number of participants included. In addition to such methodological concerns, varying cognition assessment, differing exposure estimation methods and lags, the cross-sectional design of many analyses and inconsistencies in the direction and magnitude of reported associations preclude conclusive inference to date. In a recent review [40] on a related health outcome (dementia incidence) the effects are separately calculated for studies which had a “passive” follow-up (mainly through data linkage) and those with “active” follow-up and the larger effect found for PM_2.5_ exposures in the latter studies was emphasised, stressing the need for detailed outcome assessment.

In the present study, associations between long-term exposure to specific air pollutants and cognitive function were investigated using the English Longitudinal Study of Ageing (ELSA; [33]) cohort. This cohort included adults aged 50 years and older at recruitment and applied repeated measurements to assess cognition over a 15-year period with an average number of interviews exceeding five, making the present study one of the longest such epidemiological studies with repeated measurements undertaken to date.

## Materials and methods

### Study population

ELSA is an ongoing interdisciplinary cohort study of adults aged ≥ 50 years from across England [33, 35]. ELSA was established in 2002 with biennial follow-up interviews conducted. The baseline cohort (*n* = 11,391) was drawn from households that had responded to the Health Survey for England (HSE) in 1998, 1999 and 2001. The HSE was designed to be representative of the English population living in private households [23] and ELSA is broadly representative of the English population aged 50 years and older in terms of socio-demographic characteristics [33]. The ELSA study collects information on general health, cognition, chronic disease, socioeconomic status and behaviours such as smoking and physical activity, via in-person interviews, self-completion questionnaires and nurse visits. Respondents aged 50 years and older at baseline (‘core members’) from across England were included in the present study. Interview data for a total of 11,388 ELSA respondents were provided by the National Centre for Social Research (NatCen, https://natcen.ac.uk/s/elsa-50-health-and-life). Analysis in the present study included 9,288 core members that provided an in-person baseline interview (2002–2003) and at least one subsequent in-person follow-up (2004–2017). ELSA data was provided by NatCen observing all General Data Protection Regulation (GDPR) procedures and no data was collected under the responsibility of the present study. Data provided was approved by the NatCen data release review panel and remained anonymised throughout analysis.

### Cognitive function

Cognitive test scores administered at interviews were used to measure cognitive function. ELSA participants were asked at each interview to undertake a cognitive test battery, including tests of memory and executive function [35]. To assess memory, tests of immediate and delayed word recall were conducted at every follow-up. These tests involved randomly assigning one of four 10-word lists that were read aloud to respondents, who were then asked to recite as many of the ten words as they could remember, both immediately (immediate recall) and after some time had passed and they had been involved in other cognitive tasks (delayed word recall). Previous studies have compiled a composite memory score by combining the results of these two cognitive tests [22, 24, 42]. The present study implemented the same protocol and calculated a score ranging from 0 to 20 as a composite measure of memory. Executive function was tested using an animal naming test in which respondents were asked to name as many animals as they could within one minute. The total number of animals named was used as the executive function score. Both memory and animal naming tests were included in all ELSA follow-up interviews with the exception of the 2012–13 follow-up interview which did not include the animal naming test. Tests of word recall and the animal naming test have been shown to display good construct validity both in ELSA and other cohorts [29, 44].

### Exposure to pollutants

Exposure to NO_2_, PM_10_, PM_2.5_ and ozone was estimated at the subjects’ residential postcode using the Community Multiscale Air Quality Urban (CMAQ-urban; [2]) dispersion model. A postcode in the UK provides a very fine spatial scale, as each postcode includes 14 households on average. The model incorporates the CMAQ [4] and Atmospheric Dispersion Modelling System Roads models, as well as the National and London Atmospheric Emissions Inventories (NAEI and LAEI) to estimate hourly concentrations of the pollutants at a 20 × 20 m grid level across the UK [2]. Residential postcode was made available for linkage for each ELSA participant’s residence and CMAQ-urban estimates were, therefore, averaged for each pollutant at postcode level by calculating the annual average concentration (from hourly modelled estimates) within the grid cell containing the postcode centroid. Estimates were modelled specifically at two time-points: 2004 and 2012 annual average concentrations for all postcodes across England (approximately 2.1 million postcodes). Validation of CMAQ-urban modelled concentrations against measured concentrations at daily level was conducted for London [12] and provided cross-validated R^2^ values of > 0.70 for PM and NO_2_. Validation of annual estimates derived from the CMAQ-urban model was conducted and further information is provided in Supplementary Material Figure S1 and Table S1 for the whole of the UK, where good performance in comparison with a holdout data set of measured concentrations across a national fixed-site monitoring network was observed for all pollutants, with *r* values ranging from 0.78 to 0.91 for 2004 and 0.67 to 0.90 for 2012.

To protect respondents’ identity and eliminate the possibility of post hoc identification of respondent postcodes through point-estimate combinations, postcode level annual average pollutant estimates across England were classified into categories (using deciles of the England-wide distribution for NO_2_, PM_10_ and PM_2.5_; quintiles for ozone; Supplementary Material Table S2). The corresponding mid-range concentration per pollutant category was then assigned to respondents according to the postcode of their residence as an estimate of exposure. For participant interviews conducted between 2002 and 2009 residential concentrations were assigned the 2004 estimates, whilst for interviews conducted between 2010 and 2017 residential concentrations were assigned the 2012 estimates, based on the assumption that spatial variability remains consistent in adjacent years. ELSA participants that moved home during the course of follow-up were accounted for, with assigned concentration estimates applied to the residential postcode at the time of interview.

Concentrations of NO_2_ and particulate matter (PM) are generally higher in London (whilst ozone is often lower) in comparison to the rest of England and London-dwelling ELSA participants were exposed at the highest concentrations of NO_2_ and PM through the categorisation process. Therefore, for the London-only sub-sample of ELSA respondents, the two highest deciles for NO_2_ and PM were further categorised into the lower and upper 50% of nationwide postcodes per decile, resulting in two more categories for London-dwelling participants and an increase in heterogeneity at the highest concentrations for the London-only analysis (Supplementary Material Table S3).

### Potential confounders and other covariates

Gender and educational level were measured at baseline, whilst age, smoking status and physical activity were assessed at each interview. Smoking status was classified into three categories: current smoker, former smoker or never smoker. For physical activity, respondents were asked to answer three questions, scored on a scale of 1 (“more than once a week”) to 4 (“hardly ever/never”), regarding how often they partake in vigorous, moderate and light physical activity. A weighted total was calculated to form a composite physical activity variable categorising the respondent as “very active” (1 – 2), “moderately active” (2 – 3) or “sedentary” (3 – 4) at baseline and each follow-up [11, 17]. As an indicator of socioeconomic status (SES), age at which participants left full-time education was collected at the baseline interview and coded as a categorical variable (see Table 1 for categories). The number of interviews was also adjusted for as a covariate to account for selective drop-out.

**Table 1:** Baseline descriptive statistics of demographic and interview data for ELSA respondents included in analyses of cognitive function in both the national and London sub-studies

	England-wide ELSA participants	London-wide ELSA respondents
Number of participants	8,883	768
Number of interviews provided, mean ± SD	5.70 ± 2.29	5.63 ± 2.26
Years of follow-up, mean ± SD	10.04 ± 4.56	10.10 ± 4.57
Sex		
Women, n (%)	4,897 (55.1%)	438 (57%)
Men, n (%)	3,986 (44.9%)	330 (43%)
Age at recruitment (years), mean ± SD	64.40 ± 9.75	64.40 ± 10.18
Physical activity		
Sedentary, n (%)	1,996 (22.5%)	214 (27.9%)
Moderately active, n (%)	3,938 (44.3%)	343 (44.6%)
Very active, n (%)	2,946 (33.2%)	211 (27.5%)
Smoking status		
Never smoked, n (%)	3,217 (36.2%)	283 (36.8%)
Former smoker, n (%)	4,115 (46.3%)	330 (43%)
Current smoker, n (%)	1,551 (17.5%)	155 (20.2%)
Age left full-time education (n participants)	3,825	319
14 or younger/Never went, n (%)	416 (10.9%)	40 (12.5%)
At 15, n (%)	1,363 (35.6%)	91 (28.5%)
At 16, n (%)	809 (21.2%)	51 (16%)
At 17, n (%)	333 (8.7%)	35 (11%)
At 18, n (%)	251 (6.6%)	29 (9.1%)
19 or older, n (%)	653 (17%)	73 (22.9%)

### Statistical analysis

Linear mixed-effects models were implemented to analyse the associations between exposure to ambient air pollution and repeated measurements of cognitive function. The dependent variable was the cognitive test score at each follow-up included as a continuous variable. Repeated measurements (cognitive testing at each follow-up interview) for each ELSA participant were accounted for via the inclusion of a random intercept per individual. Single pollutant models were fitted initially, with concentration estimates for the previous interview (or baseline for the baseline interview) included as the exposure variable in order to assess the long-term effects of pollutant exposure on cognition. Two-pollutant models were also applied for pollutants not highly correlated (*r* < 0.7; Supplementary Material Table S4). The models were adjusted for potential confounders: age (years) as a time-varying continuous covariate, smoking status (current, former and never smokers) and physical activity (sedentary, moderately active, very active) were included as time-varying categorical variables, whilst gender as recorded at baseline (categorical; females as the reference category) and the number of interviews provided (continuous). An additional sensitivity analysis was conducted in which the number of interviews was excluded as a covariate. Age at which the participant left education was included as a categorical variable in a separate sensitivity analysis as this variable had a large number of missing values and was only available for 3,825 participants. A total of 84 ELSA participants reported to still be in full-time education. As it is uncertain how additional ongoing education after the age of 50 years old would influence SES within a short time period, we chose to exclude these 84 individuals from the corresponding analyses. We applied the same models using the modified exposure categories for the ELSA participants residing in London. ELSA respondents with complete covariate information were included in analyses (8,883 in the England-wide study; 768 in the London sub-study). All analyses were conducted in R version 4.2.1 [30].

## Results

Baseline summary statistics for ELSA respondents included in analyses are provided in Table 1. In total, 8,883 participants provided an average number of 5.7 interviews including cognitive assessment, across more than 10 years of follow-up. Age, number of interviews and follow-up time were similar between the England and London samples. There were relatively more women in the London sample, more current smokers and more subjects with a sedentary lifestyle.

Descriptive statistics (by 10-year age groups) for baseline cognitive test scores of memory and executive function are provided in Table 2 for both the England and London sample populations. Across both samples, test performance was inversely associated with baseline age, with those in the oldest age group scoring more than 50% and 36% lower than those in the youngest age on average in memory and executive function tests, respectively in the England wide sample, whilst the contrast observed in London residents was smaller. A wide range was observed across all ages for both test scores, with most age groups represented by at least one individual scoring zero. Older respondents also displayed steeper rates of decline (Supplementary Material; Figure S2 and Figure S3).

**Table 2:** Descriptive statistics of baseline cognitive test scores provided by ELSA respondents included in analyses of cognitive function across England and London, separated by baseline age group

Baseline age (years)	n	Min	1st Quartile	Mean ± SD	Median	3rd Quartile	Max
England-wide sample (*n* = 8,883)							
Composite Memory Score (0 – 20)							
50–59	3,401	0	9	11.0 ± 3.1	11	13	20
60–69	2,764	0	8	9.8 ± 3.2	10	12	20
70–79	1,959	0	6	8.3 ± 3.3	8	11	18
80–89	725	0	4	6.8 ± 3.2	7	9	16
90 +	34	0	3	5.2 ± 2.8	5	8	10
Executive Function Score							
50–59	3,401	0	18	21.6 ± 6.3	21	26	50
60–69	2,764	0	16	19.8 ± 6.0	20	23	48
70–79	1,959	0	14	17.7 ± 5.5	17	21	49
80–89	725	0	12	16.0 ± 5.5	16	19	37
90 +	34	5	10	13.8 ± 4.1	14	17	24
London-wide sample (*n* = 768)							
Composite Memory Score (0 – 20)							
50–59	308	1	9	10.6 ± 3.5	11	13	20
60–69	232	0	8	9.8 ± 3.1	10	12	16
70–79	150	0	6	8.6 ± 3.3	9	11	16
80–89	71	0	5	7.0 ± 3.1	7	9	14
90 +	7	4	6	7.1 ± 2.0	8	8	10
Executive Function Score							
50–59	308	0	17	20.9 ± 7.0	21	25	50
60–69	232	5	15	19.0 ± 6.4	18	23	40
70–79	150	1	13	17.5 ± 5.8	17	21	35
80–89	71	1	11	15.6 ± 6.1	15	20	37
90 +	7	9	11	14.4 ± 4.9	14	16	24

Mean baseline annual concentration estimates for each pollutant assigned to ELSA respondents at their residence are provided in Table 3. In the London-wide sample, mean assigned baseline concentrations were higher for NO_2_ (by 65.9%), PM_10_ (by 10.5%) and PM_2.5_ (by 37.9%) than those in the England-wide sample, whilst the mean London concentration was lower for ozone by 43.2%. Good spatial correlation between early (2004–2009) and late (2010–2017) exposure window assignment was observed for all pollutants.

**Table 3:** Baseline CMAQ-urban modelled pollutant concentrations (µg/m^3^) linked to ELSA respondents’ postcodes of residence included in the analyses for both the England and London sample populations

Pollutant	Baseline mean ± SD (µg/m^3^)
England-wide respondents	
NO_2_	25.58 ± 13.67
PM_10_	19.20 ± 10.57
PM_2.5_	12.15 ± 4.45
Ozone	46.88 ± 13.61
London-wide respondents	
NO_2_	42.45 ± 14.69
PM_10_	21.21 ± 8.23
PM_2.5_	16.76 ± 5.88
Ozone	26.62 ± 10.76

**Fig. 1:**
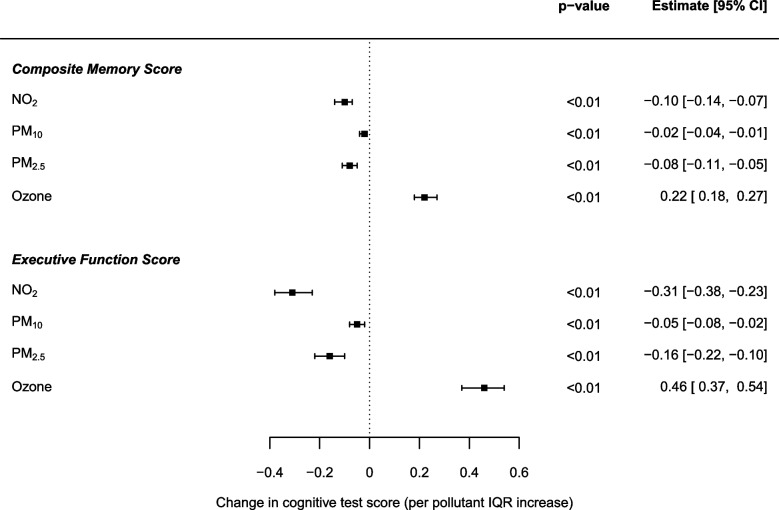
Change in the composite memory and executive function scores per IQR increase in NO_2_ (13.05 μg/m^3^), PM_10_ (3.35 μg/m^3^), PM_2.5_ (2.70 μg/m^3^) and ozone (15.05 μg/m^3^) concentrations: single pollutant mixed-effects model effect estimates for 8,883 ELSA respondents across England adjusted for age, gender, number of interviews, smoking status and physical activity

Increased long-term exposures to NO_2_, PM_10_ and PM_2.5_ were associated with decreased scores in both memory and executive function tests, whilst ozone exposure was associated with a protective effect (Fig. 1). Interquartile range (IQR) increases in NO_2_ (13.05 μg/m^3^), PM_10_ (3.35 μg/m^3^), PM_2.5_ (2.70 μg/m^3^) and ozone (15.05 μg/m^3^) were associated with a decrease of -0.10 [95% CI: -0.14, -0.07], -0.02 [-0.04, -0.01] and -0.08 [-0.11, -0.05], respectively, in composite memory score. Similar decreases were observed for executive function score of -0.31 [-0.38, -0.23], -0.05 [-0.08, -0.02] and -0.16 [-0.22, -0.10], respectively. In contrast, increasing ozone exposure per IQR was associated with an increase in composite memory score by 0.22 [0.18, 0.27] and in executive function by 0.46 [0.37, 0.54]. In order to test potential non-linearity in the association between ozone exposure and cognitive function, additional models were fitted adjusting for ozone as a categorical variable (see Supplementary Table S2) with no indication of a non-linear trend observed between ozone and cognition for both test scores (data not presented here). The direction and magnitude of effect estimates remained robust with the exclusion of number of respondent interviews as a covariate, with some adverse effects strengthened.

The results observed in two-pollutant models did not substantially alter the main findings of the single pollutant models, with estimated changes in cognitive test score differing by 3% or less across all multi-pollutant models (Supplementary Material Table S7). Changes of 0.01 to 0.03 in test score effect estimates for models investigating the effects of PM_10_ exposure did however provide marginally non-statistically significant results when adjusting for NO_2_ and ozone in composite memory score and NO_2_ in executive function score.

**Fig. 2:**
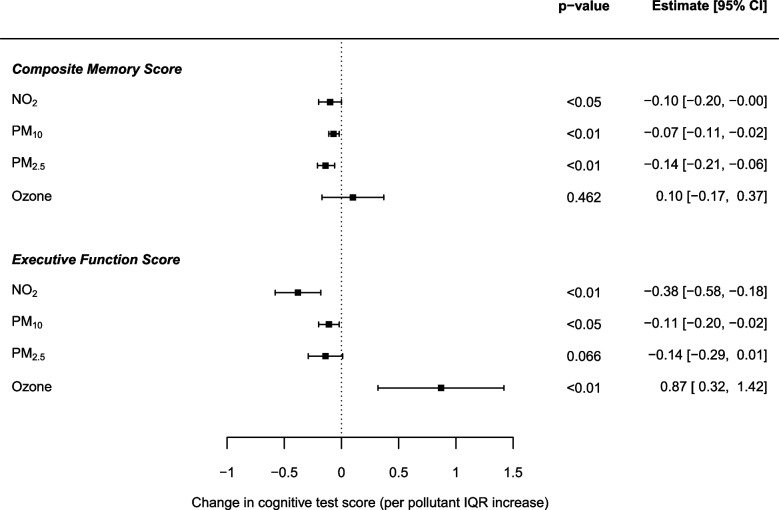
Change in composite memory and executive function scores per IQR increase in NO_2_ (11.10 μg/m^3^), PM_10_ (2.35 μg/m^3^), PM_2.5_ (2.50 μg/m^3^) and ozone (21.25 μg/m^3^) concentrations: single pollutant mixed-effects model effect estimates for 768 ELSA participants that were residents of London, adjusted for age, gender, number of interviews, smoking status and physical activity

In London-dwelling ELSA respondents, the direction of effect of each pollutant across both cognitive test scores remained the same as for the England-wide sample, although the magnitude of those reaching the nominal level of statistical significance increased (Fig. 2).

**Fig. 3:**
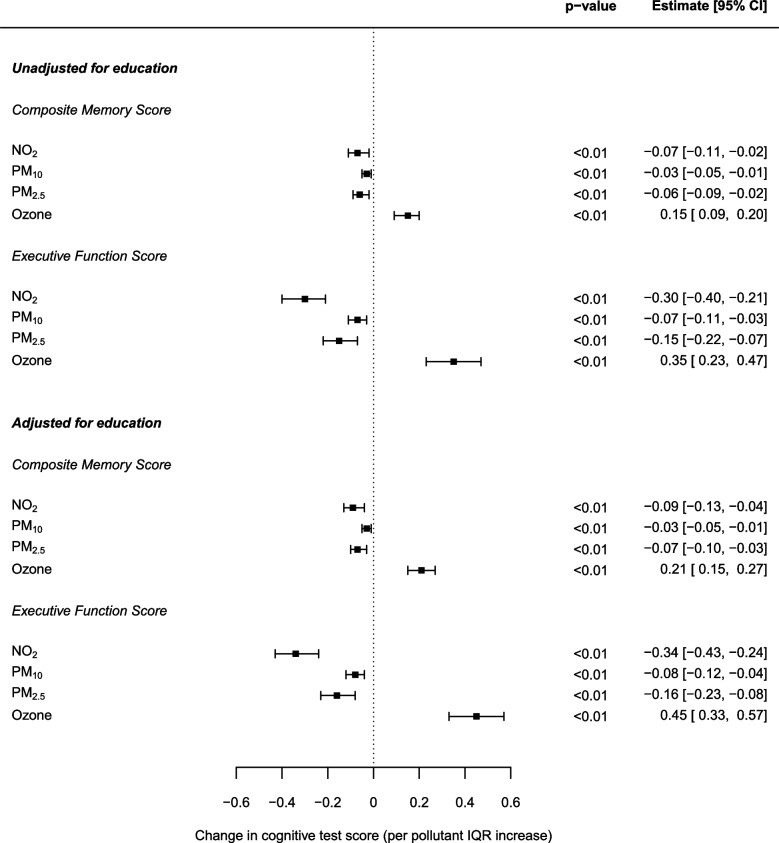
Change in the composite memory and executive function scores per IQR increase in NO_2_ (13.05 μg/m^3^), PM_10_ (3.35 μg/m^3^), PM_2.5_ (2.70 μg/m^3^) and ozone (15.05 μg/m^3^) concentrations: single pollutant mixed-effects model effect estimates for 3,825 ELSA respondents across England adjusted for age, gender, number of interviews, age at which full time education was left, smoking status and physical activity

The additional inclusion of education information (age at which left full-time education) as a further covariate to the models described in the main analysis was conducted for a sub-sample of the ELSA cohort data made available to the present study (*n* = 3,825). Figure 3 displays the results of comparable models to those applied for the whole cohort (shown in Fig. 1) alongside models adjusting for education in the same sample. Pollutant effect estimates remained very similar both to that of the main analysis and between the two models (adjusting and not adjusting for education).

In a model including only all available confounders (Supplementary Material Table S6), increasing age was associated with poorer cognitive test performance, whereas respondents providing a greater number of follow-up interviews performed better. Male participants displayed better executive function test performance (0.34 [0.04, 0.65]), but exhibited worse composite memory scores in comparison to female respondents (-0.69 [-0.83, -0.55]). Those reporting to be sedentary at baseline performed worse across both cognitive measures in comparison to those reporting to be moderately active (-0.49 [-0.58, -0.40] for composite memory score and -0.88 [-1.06, -0.70] for score on the test of executive function), whilst those reporting to be very active were indicated to perform better across both tests. The observed beneficial effects of being a non-smoker compared to being a current or former smoker did not reach the nominal level of statistical significance for scores in either cognitive measure. Increasing score on both the memory and executive function tests was associated with increasing years in full-time education, which reached a 2.12 [1.92, 2.33] units increase in memory score and a 3.78 [3.34, 4.22] units increase in the executive function score for participants who remained in full-time education until 19 years of age or older, compared to those who left full time education at 15 years old.

## Discussion

The present study assessed cognitive test performance in English older adults in relation to long-term air pollution exposure at the residential address. The follow-up period of 15 years and the large number of repeated measurements make the present study unique in terms of design and data availability. Increasing exposure to NO_2_, PM_10_ and PM_2.5_ was consistently found to be associated with decreased memory and executive function test performance, whilst ozone showed the opposite effect. The results remained similar in the analysis including residents of London only, for whom exposure to NO_2_ and PM was higher. As an illustrative example, the decline in memory and executive function scores per IQR increase in long-term NO_2_ exposure was found equivalent to ageing by about 1.5 and 4 years (combining results from Fig. 1 and Table 3) respectively.

Many of the studies reporting on the association between exposure to pollutants and executive function used the animal naming test as a test of semantic fluency and based their analyses on cross-sectional data. In a cross-sectional study, Tzivian et al. [38] analysed 4,050 individuals in Germany and reported similar findings to the present study with a -0.07 [-0.11, -0.03] decrease in score on the animal naming test estimated per 1.44 µg/m^3^ increase in PM_2.5_ exposure, as well as suggestive (not statistically significant) evidence for adverse associations with NO_2_ and PM_10_. Additionally, Salinas-Rodriguez et al. [31] cross-sectionally analysed data from 7,986 Mexican individuals, reporting a statistically significant reduction in animals named (-0.72 [-1.05, -0.40]) per 10 µg/m^3^ increase in PM_2.5_ exposure. Tonne et al. [37] found increasing exposure to PM_2.5_ to be associated with slightly reduced performance on a cognitive test assessing reasoning ability (a mental process of executive function) in a cross-sectional investigation of 3,414 civil servants in London (mean age 61 years), UK, with small associations reported for PM_2.5_ exposure at several lag structures (e.g., − 0.043 [− 0.082, − 0.004]; per yearly lag4 increase of 1.3 µg/m^3^), whilst no statistically significant associations were reported between PM and performance on the animal naming test. In the longitudinal component of the study including one follow-up cognitive test taken approximately five years later by 2,867 individuals, the authors report non-statistically significant associations between PM exposure and declining reasoning and semantic fluency test performance. Cullen et al. [9] found small and inconsistent associations between PM_10_ and NO_2_ exposure and reasoning in a cross-sectional analysis of 86,759 participants of the UK Biobank cohort (mean age 58 years), finding no association for PM_2.5_. No associations were found for exposure to any pollutant when assessing decline in test scores of executive function in a longitudinal analysis of 2,913 participants that returned for a follow-up visit within 2.8 years.

In terms of memory, previous studies assessing performance on memory tests in relation to air pollution exposure have produced generally supportive evidence for an adverse association but inconsistencies in results exists between studies [36]. Tonne et al. [37] report non-statistically significant adverse associations between PM and performance on tests of memory in the Whitehall II cohort in both cross-sectional and longitudinal analyses. Cullen et al. [9] observed no associations between NO_2_ or PM with performance on tests of numeric or prospective memory but did find increasing NO_2_ exposure (by 1 µg/m^3^) to be associated with reduced performance on a task of visuospatial memory in a cross-sectional analysis of the UK Biobank cohort. The longitudinal analysis undertaken (including 2,913 individuals with one follow-up) reported no statistically significant associations between memory test performance and exposure to any pollutant. Ailshire and Crimmins [1] observed non-linear associations between performance in the same tests of memory as used in the present study and exposure to PM_2.5_ in a cross-sectional analysis of a nationally representative sample of 13,996 respondents to the Health and Retirement Study of the US in 2004.

Of the limited number of studies including repeated measures to date, Park et al. [25] provide the largest investigation in terms of participants and follow-up (*n* = 398,889 older adults in Seoul, South Korea), with 64,836 completing at least five MMSE Mini-Mental State Exam (MMSE) tests measuring global cognition. The data were constructed from administrative data with a mean follow-up time of 4.2 years, potentially limiting the ability of the study to assess the long-term effects of air pollution on cognitive test performance. The study reported increasing NO_2_ and PM_10_ to be associated with greater decreases in MMSE score, with the opposite effect observed for ozone. No domain-specific tests were implemented in the study.

The positive association between ozone and cognitive function observed in the present study may be partly due to the negative correlation between ozone and NO_2_/PM (Supplementary Material Table S4). There was no indication of non-linearity in the relationship between ozone exposure and cognitive test scores when ozone was included as a categorical variable. The limited number of studies to date have produced mixed results. Several studies report an adverse association between ozone and global or domain-specific cognitive function [6, 15, 18, 20], although none of these studies were conducted in the UK. Notably, Lo et al. [20] observed adverse associations between long-term exposure to ozone (as well as co-exposure of ozone and PM_10_) with scores of global cognition across four repeated measures in a Taiwanese cohort, although just 952 of the original sample of 2,241 completed all follow-up tests. Shin et al. (2019) observed improved recall test performance with increasing exposure to ozone in a cross-sectional analysis of 2,896 Korean adults. Inconsistent direction of associations between ozone exposure and tests of memory were also reported by Gatto et al. [16] in a cross-sectional analysis of 1,496 individuals in Los Angeles, USA. The inconsistencies in the direction of association between cognitive test performance and ozone exposure to date highlight the necessity for further investigation into the effects of ozone on cognition.

In order to fully elucidate potentially adverse cognitive effects of air pollution, further study into the underlying biological pathways and mechanisms through which air pollution may contribute to cognitive decline is required alongside the expanding epidemiological work. Translocation of inhaled particles from the lung to the brain via the bloodstream provides one possible pathway through which particulate matter may affect cognition, as well as inhalation through the nose and transportation to the olfactory bulb via olfactory nerves. Evidence for such pathways is currently limited and further experimental studies are required [8].

The association of cognitive tests with the confounders included in the present study were largely in the expected direction and magnitude [10, 34, 43] adding confidence in the validity of our results. Poorer cognitive test performance was associated with increasing age, fewer years of education, current smoking and a sedentary lifestyle. Male respondents performed worse on tests of memory in comparison to women but attained higher scores on the executive function test.

Strengths of the present study include the long-term follow-up period (> 15 years) combined with a large number of cohort participants and repeated measurements for memory and executive function tests (approximately six per person on average). The majority of previous studies investigating the association between cognitive function and air pollution exposure rely on cross-sectional analyses and those with a longitudinal component generally included a markedly smaller number of subjects, shorter follow-up periods and one or few follow-up tests. Another advantage of the present study is the use of a validated model to assess exposure to air pollutants at a fine spatial scale (a postcode in the UK includes on average 14 households) and for sufficiently long periods compared to published studies to date [39]. A further advantage of the ELSA cohort is that it is broadly representative of the English population as it is based on the Health Survey for England [33], thus reducing the potential for selection bias. A previous paper [41] reported from the same data on the effect of exposure to pollutants on the incidence of dementia in ELSA respondents and found suggestive effects of PM on increased risk. However, Wood et al. [41], although reporting broadly consistent results to the present analysis, addressed the incidence of an event and had inherently smaller statistical power.

The present study has some limitations. One limitation was that the exposure data were categorised to maintain that participant postcodes and identity could not be identified post hoc. This potentially limited the power of detecting associations between exposure and cognitive function as it led to larger measurement error (e.g., [32]) and potentially to underestimated associations [3]. However, the contrast in exposures was preserved since the categorisation was based on deciles of postcode distributions across England and further refined for London.

Another potential limitation was the lack of adjustment for anthropometric, comorbidity and further socioeconomic data for the participants which may be potential confounders or effect modifiers, such as alcohol intake, environmental tobacco smoke exposure, body mass index (BMI), sleep, comorbidities, medication use and diet, as previous studies have found these to be potential risk factors associated with impaired cognitive performance in later life (e.g., [5, 13, 14, 26]). However, these outcome determinants may not be confounders of the air pollution-cognition association, as is demonstrated in the present analysis where the results did not change with adjustment for education level and such findings have been reported in previous studies [31, 37]. The relationship between air pollution exposure and BMI remains unclear, whilst studies assessing comorbidities (such as cardiovascular disease and stroke) as effect modifiers of pollutant exposures and cognitive impairment have provided inconsistent results [10].

A further limitation is the fact that shorter follow-up, i.e., drop-out from the study, is associated with the outcome. This selective drop-out leads to longer follow-up for those who perform better in cognitive tests and may be linked to an underestimation of the association between exposure to pollutants and cognition. In the present analysis the number of interviews provided was adjusted for. However, further methods of analysis accounting for this may be explored in the future.

Further work may also aim to investigate potential variation in the observed associations between pollutant exposure and cognitive test performance by season. The aim of the present study was to assess the effects of long-term exposure on cognitive function, however the inclusion of exposure data at a finer temporal resolution may provide further insight into the relationship between seasonally variant pollutants (such as ozone) and cognitive test performance.

In conclusion, the present study provides evidence of an adverse association between long-term exposure to NO_2_, particulate matter pollution and longitudinal performance in the cognitive domains of memory and executive function. The study was based on long-term follow-up, several repeated biannual cognitive function assessments and modelling of longitudinal air pollution exposure at a fine spatial scale. The decline in memory and executive function scores per IQR increase in long-term NO_2_ exposure was found equivalent to ageing by approximately 1.5 and 4 years, respectively. More research should be undertaken in the future in different locations with varying environmental and demographic conditions, as well as using detailed cohort data with longer follow-up and more detailed assessment of individual conditions.

As exposure to air pollution is one of the few factors related to cognitive decline that is modifiable at the population level, these results can lead to the prevention of associated cognitive decline and dementia incidence, major public health problems in ageing populations.

### Supplementary Information

**Supplementary Material 1.**

## Data Availability

The English Longitudinal Study of Ageing (ELSA) was developed by a team of researchers based at University College London, the Institute for Fiscal Studies and the National Centre for Social Research. The data are linked to the UK Data Archive and freely available through the UK data services and can be accessed at: https://beta.ukdataservice.ac.uk/datacatalogue/studies/study?id=5050 (accessed on 10 November 2017).

## Abbreviations

CMAQ: Community Multiscale Air Quality model
CMAQ-urban: Community Multiscale Air Quality Urban dispersion model
COMEAP: Committee on the Medical Effects of Air Pollutants
ELSA: English Longitudinal Study of Ageing
LAEI: London Atmospheric Emissions Inventory
NAEI: National Atmospheric Emissions Inventory
NatCen: National Centre for Social Research
NO_2_: Nitrogen dioxide
O_3_: Ozone
PM: Particulate matter
PM_10_: Particulate matter up to 10 µm in diameter
PM_2.5_: Particulate matter up to 2.5 µm in diameter
