# Supplementary material for: Exposure to ambient air pollution and cognitive function: an analysis of the English Longitudinal Study of Ageing cohort
Source: Environ Health. 2024 Apr 5;23:35. doi: 10.1186/s12940-024-01075-1 (PMC10996194; doi:10.1186/s12940-024-01075-1)
Supplement: Supplementary file 1 — Supplementary Material 1. [file 12940_2024_1075_MOESM1_ESM.docx]

**Supplementary Material**


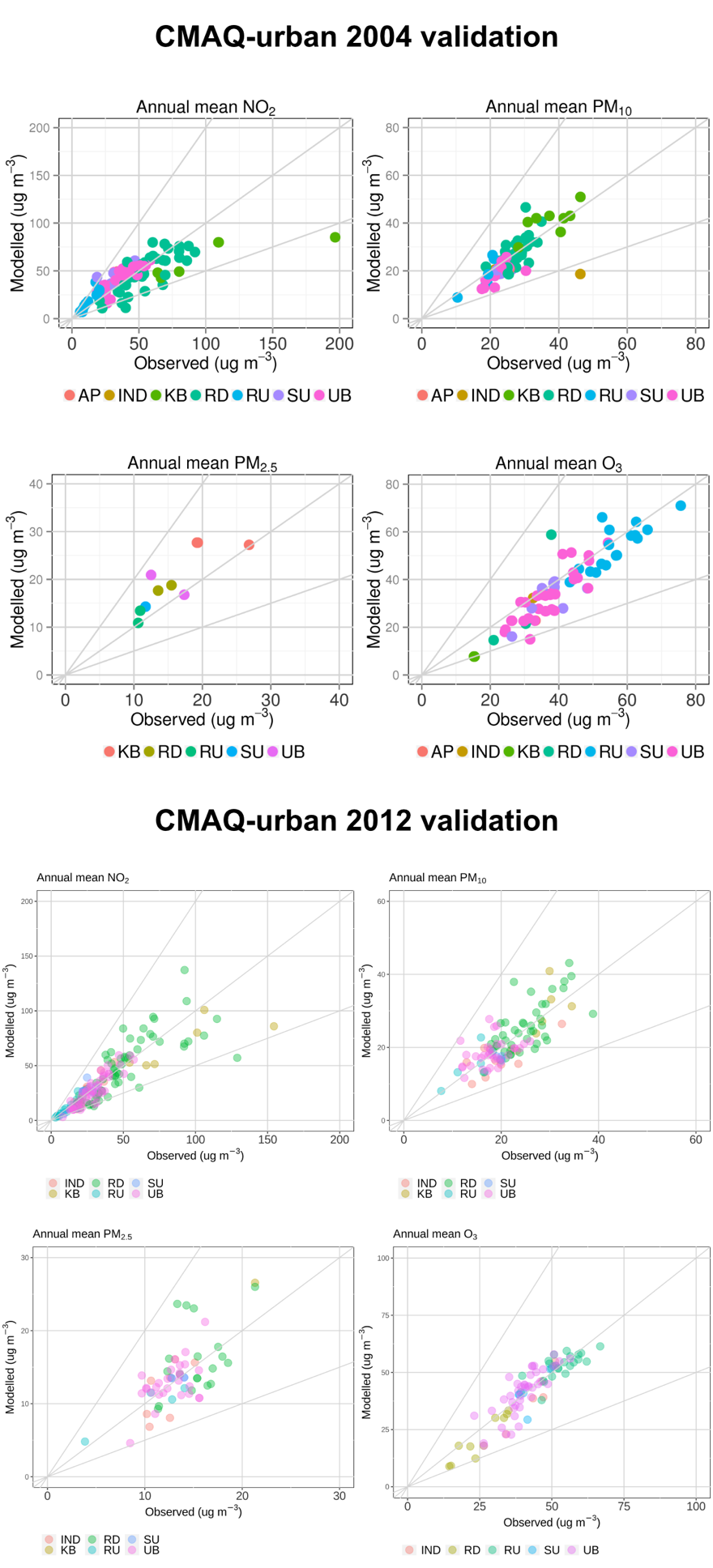


**Figure S1.** CMAQ-urban 2004 and 2012 model validation. Annual average observed (monitor concentrations) plotted against annual average modelled concentrations at the 20x20m grid cell in which the monitor is located. Monitor site type denoted by colour. **AP**: airport; **IND**: industrial; **KB**: kerbside; **RD**: roadside; **RU**: rural; **SU**: suburban; **UB**: urban background.

**Table S1.** CMAQ-urban 2004 and 2012 model performance and validation in comparison to measured concentrations of NO_2_, PM_10_, PM_2.5_ and ozone.

|  | **2004** | | |  | **2012** |  |
| --- | --- | --- | --- | --- | --- | --- |
| **Pollutant** | **N Monitor Sites** | **RMSE** | **r** | **N Monitor Sites** | **RMSE** | **r** |
| **NO_2_** | 122 | 15.32 | 0.78 | 155 | 13.32 | 0.86 |
| **PM_10_** | 88 | 5.13 | 0.78 | 102 | 4.50 | 0.78 |
| **PM_2.5_** | 10 | 5.04 | 0.82 | 63 | 3.17 | 0.67 |
| **Ozone** | 69 | 7.11 | 0.91 | 87 | 5.54 | 0.90 |

**Table S2**. Annual average concentrations of NO_2_, PM_10_, PM_2.5_ and ozone for 2004 estimated by CMAQ-urban categorised into England-wide deciles per pollutant (quintiles for ozone) and the total number of ELSA participants (n = 8,883) assigned each concentration at baseline.

|  | **Categorical exposure variable** | **Range**  **(µg/m^3^)** | **Mid-range concentration (µg/m^3^)** | **ELSA participants at baseline** |
| --- | --- | --- | --- | --- |
| **NO_2_** |  |  |  |  |
|  | **1** | 1.8-13.2 | 7.5 | 1,002 |
|  | **2** | 13.2-17.2 | 15.2 | 1,043 |
|  | **3** | 17.2-19.9 | 18.55 | 912 |
|  | **4** | 19.9-22.4 | 21.15 | 932 |
|  | **5** | 22.4-25.5 | 23.95 | 1,095 |
|  | **6** | 25.5-28.5 | 27 | 1,030 |
|  | **7** | 28.5-32.5 | 30.5 | 965 |
|  | **8** | 32.5-37.5 | 35 | 953 |
|  | **9** | 37.5-44.8 | 41.15 | 674 |
|  | **10** | 44.8-119.1 | 81.95 | 277 |
| **PM_10_** |  |  |  |  |
|  | **1** | 8.2-14 | 11.1 | 1,008 |
|  | **2** | 14-15.3 | 14.65 | 952 |
|  | **3** | 15.3-16.1 | 15.7 | 1,021 |
|  | **4** | 16.1-16.7 | 16.4 | 980 |
|  | **5** | 16.7-17.3 | 17 | 1,115 |
|  | **6** | 17.3-17.9 | 17.6 | 950 |
|  | **7** | 17.9-18.7 | 18.3 | 897 |
|  | **8** | 18.7-19.8 | 19.25 | 712 |
|  | **9** | 19.8-22.4 | 21.1 | 683 |
|  | **10** | 22.4-94.7 | 58.55 | 565 |
| **PM_2.5_** |  |  |  |  |
|  | **1** | 4.1-8.5 | 6.3 | 1,036 |
|  | **2** | 8.5-10.5 | 9.5 | 1,087 |
|  | **3** | 10.5-11.4 | 10.95 | 868 |
|  | **4** | 11.4-11.9 | 11.65 | 937 |
|  | **5** | 11.9-12.3 | 12.1 | 991 |
|  | **6** | 12.3-12.7 | 12.5 | 986 |
|  | **7** | 12.7-13.2 | 12.95 | 1,077 |
|  | **8** | 13.2-14 | 13.6 | 901 |
|  | **9** | 14-15.2 | 14.6 | 675 |
|  | **10** | 15.2-48.3 | 31.75 | 325 |
| **Ozone** |  |  |  |  |
|  | **1** | 0-37 | 18.5 | 937 |
|  | **2** | 37-42.5 | 39.75 | 2,056 |
|  | **3** | 42.5-47.1 | 44.8 | 1,904 |
|  | **4** | 47.1-52.9 | 50 | 1,973 |
|  | **5** | 52.9-79.7 | 66.3 | 2,013 |

**Table S3**. Annual average concentrations of NO_2_, PM_10_, PM_2.5_ and ozone for 2004 estimated by CMAQ-urban categorised into England-wide deciles per pollutant (quintiles for ozone; deciles 9 and 10 expanded for NO2, PM10 and PM2.5) and the total number of London-dwelling ELSA participants (n = 769) assigned each concentration at baseline.

|  | **Categorical exposure variable** | **Range**  **(ug/m^3^)** | **Mid-range concentration (ug/m^3^)** | **ELSA participants at baseline** |
| --- | --- | --- | --- | --- |
| **NO_2_** |  |  |  |  |
|  | **5** | 22.4-25.5 | 23.95 | 8 |
|  | **6** | 25.5-28.5 | 27 | 32 |
|  | **7** | 28.5-32.5 | 30.5 | 50 |
|  | **8** | 32.5-37.5 | 35 | 274 |
|  | **9.1** | 37.5-40.5 | 39 | 104 |
|  | **9.2** | 40.5-44.8 | 42.65 | 116 |
|  | **10.1** | 44.8-50.3 | 47.55 | 113 |
|  | **10.2** | 50.3-119.1 | 84.7 | 72 |
| **PM_10_** |  |  |  |  |
|  | **4** | 16.1-16.7 | 16.4 | 13 |
|  | **5** | 16.7-17.3 | 17 | 22 |
|  | **6** | 17.3-17.9 | 17.6 | 82 |
|  | **7** | 17.9-18.7 | 18.3 | 170 |
|  | **8** | 18.7-19.8 | 19.25 | 188 |
|  | **9.1** | 19.8-20.9 | 20.35 | 110 |
|  | **9.2** | 20.9-22.4 | 21.65 | 98 |
|  | **10.1** | 22.4-24.9 | 23.65 | 54 |
|  | **10.2** | 24.9-94.7 | 59.8 | 32 |
| **PM_2.5_** |  |  |  |  |
|  | **5** | 11.9-12.3 | 12.1 | 7 |
|  | **6** | 12.3-12.7 | 12.5 | 13 |
|  | **7** | 12.7-13.2 | 12.95 | 37 |
|  | **8** | 13.2-14 | 13.6 | 131 |
|  | **9.1** | 14-14.5 | 14.25 | 173 |
|  | **9.2** | 14.5-15.2 | 14.85 | 117 |
|  | **10.1** | 15.2-17 | 16.1 | 201 |
|  | **10.2** | 17-48.3 | 32.65 | 90 |
| **Ozone** |  |  |  |  |
|  | **1** | 0-37 | 18.5 | 488 |
|  | **2** | 37-42.5 | 39.75 | 229 |
|  | **3** | 42.5-47.1 | 44.8 | 52 |

**Table S4.** Spearman correlation coefficients between baseline CMAQ-urban modelled pollutant concentrations assigned to ELSA respondents.

|  | **Spearman Correlation Coefficient** | | | |
| --- | --- | --- | --- | --- |
|  | England-wide sample | | | |
|  | **NO_2_** | **PM_10_** | **PM_2.5_** | **Ozone** |
| **NO_2_** |  |  |  |  |
| **PM_10_** | 0.53 |  |  |  |
| **PM_2.5_** | 0.85 | 0.70 |  |  |
| **Ozone** | -0.89 | -0.43 | -0.79 |  |
|  | London-wide sample | | | |
| **NO_2_** |  |  |  |  |
| **PM_10_** | 0.90 |  |  |  |
| **PM_2.5_** | 0.91 | 0.96 |  |  |
| **Ozone** | -0.74 | -0.70 | -0.71 |  |

**
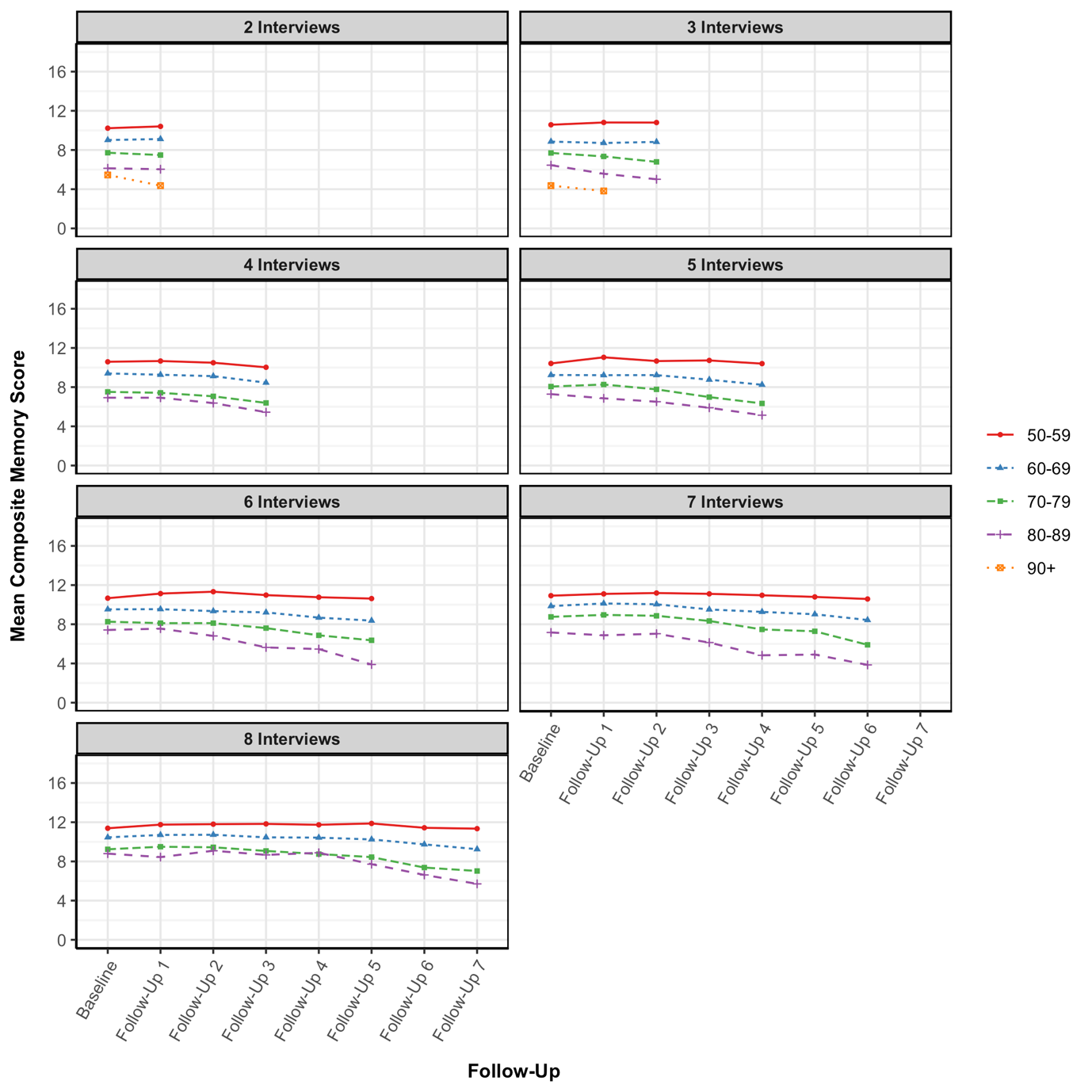
**

**Figure S2**. Mean composite memory score performance for ELSA respondents by number of interviews. Mean scores calculated where at least 10 ELSA respondents in each age category provided the given number of interviews.

**
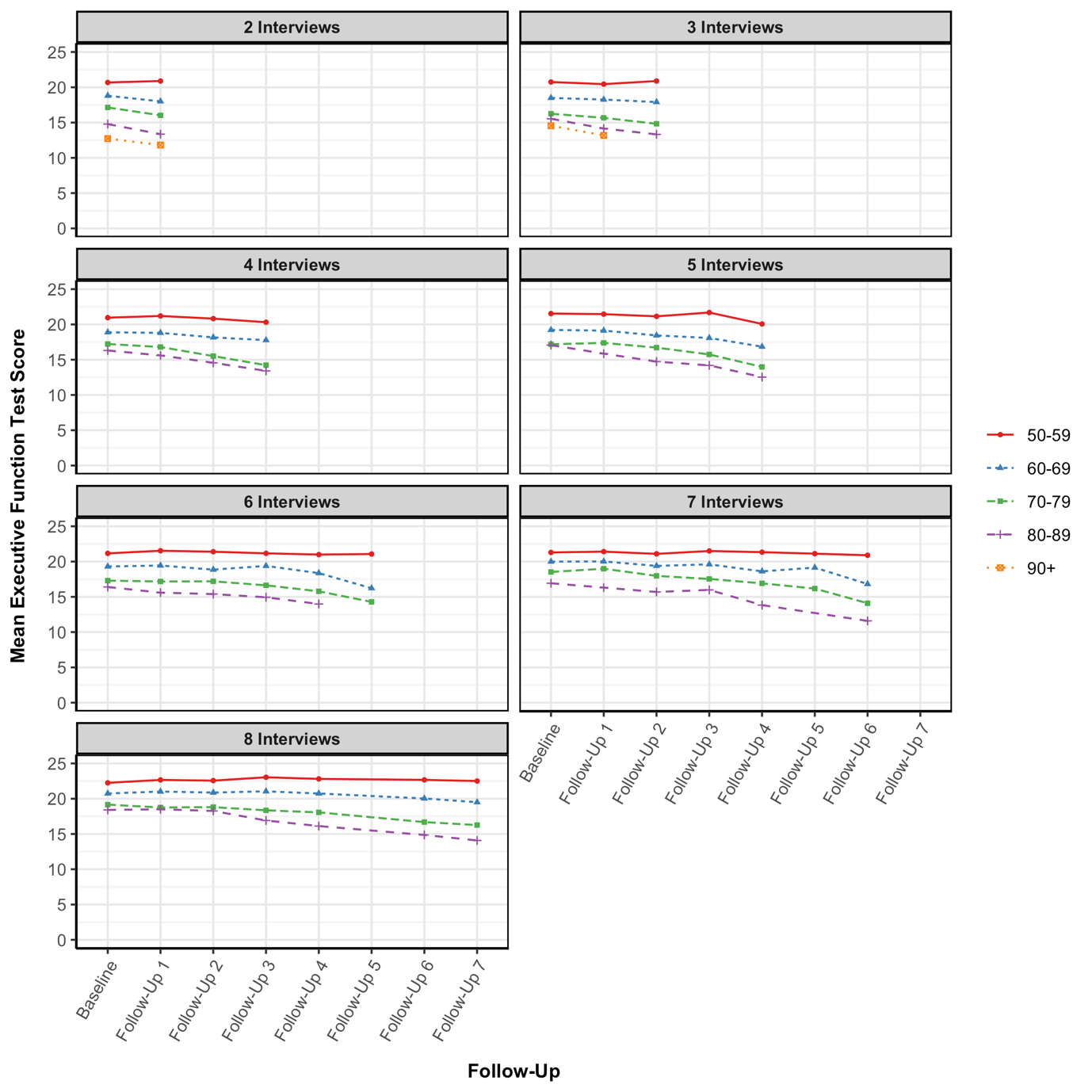
**

**Figure S3**. Mean animal naming test of executive function performance for ELSA respondents by number of interviews. Mean scores calculated where at least 10 ELSA respondents in each age category provided the given number of interviews.

**Table S5.** Mean baseline CMAQ-urban modelled pollutant concentrations assigned to ELSA respondents by 10-year baseline age group.

|  | **Mean Baseline Concentration (μg/m^3^)** | | | | | | | | |
| --- | --- | --- | --- | --- | --- | --- | --- | --- | --- |
|  | **England-wide sample** | | | | **London sample** | | | | |
| **Baseline age group** | **NO_2_** | **PM_10_** | **PM_2.5_** | **Ozone** | **NO_2_** | **PM_10_** | **PM_2.5_** | **Ozone** |  |
| **50-59** | 25.8 | 18.9 | 12.2 | 46.5 | 41.8 | 21.0 | 16.6 | 27.1 |  |
| **60-69** | 25.6 | 19.4 | 12.2 | 46.8 | 44.4 | 21.6 | 17.3 | 25.6 |  |
| **70-79** | 25.3 | 19.3 | 12.0 | 47.5 | 41.3 | 21.3 | 16.5 | 26.5 |  |
| **80-89** | 25.1 | 19.6 | 12.1 | 47.3 | 41.8 | 21.0 | 16.4 | 27.7 |  |
| **90+** | 25.2 | 18.4 | 12.6 | 45.9 | 33.9 | 18.6 | 14.3 | 32.1 |  |

**Table S6.** Mixed-effects model regression coefficients and confidence intervals for all confounders in ELSA respondents in the England-wide sample population for each cognitive test: change in the test score per unit change in the confounder (95% Confidence Interval).

| **Covariate** | **Composite Memory** | **Executive Function** |
| --- | --- | --- |
| Age (years) | -0.07 [-0.07, -0.06] | -0.07 [-0.08, -0.06] |
| Number of interviews | 0.35 [0.24, 0.46] | 0.68 [0.44, 0.92] |
| Gender |  |  |
| *Female* | *Reference category* | *Reference category* |
| *Male* | -0.69 [-0.83, -0.55] | 0.34 [0.04, 0.65] |
| Baseline physical activity |  |  |
| *Sedentary* | -0.49 [-0.58, -0.40] | -0.88 [-1.06, -0.70] |
| *Moderately active* | *Reference category* | *Reference category* |
| *Very active* | 0.21 [0.14, 0.29] | 0.40 [0.24, 0.55] |
|  |  |  |
| Baseline smoking status |  |  |
| *Never smoked* | *Reference category* | *Reference category* |
| *Former smoker* | -0.06 [-0.19, 0.07] | 0.06 [-0.22, 0.34] |
| *Current smoker* | -0.11 [-0.31, 0.09] | -0.13 [-0.57, 0.31] |
|  |  |  |
| Age at which left full-time education (n = 3,825) |  |  |
| *14 or younger/Never went* | -0.90 [-1.14, -0.66] | -1.68 [-2.20, -1.15] |
| *At 15* | *Reference category* | *Reference category* |
| *At 16* | 0.96 [0.77, 1.15] | 1.34 [0.93, 1.75] |
| *At 17* | 1.28 [1.02, 1.54] | 2.03 [1.47, 2.59] |
| *At 18* | 1.44 [1.15, 1.73] | 2.50 [1.87, 3.13] |
| *19 or older* | 2.12 [1.92, 2.33] | 3.78 [3.34, 4.22] |
|  |  |  |

**Table S7.** Change in composite memory and executive function scores per IQR increase in NO_2_, PM_10_ and ozone concentrations: results from multi-pollutant mixed-effects model effect estimates for 8,883 ELSA participants from across England; adjusted for age, gender, number of interviews, smoking status and physical activity.

| **Main effect pollutant** | **Co-pollutant** | **Change in cognitive test score per IQR increase in main effect pollutant [95% CI]** | **P value** | **Main effect pollutant IQR** |
| --- | --- | --- | --- | --- |
| Composite Memory Score | | | | |
| NO_2_ | PM_10_ | -0.09 [-0.13, -0.06] | <0.01 | 13.05 |
| PM_10_ | NO_2_ | -0.01 [-0.03, 0.00] | 0.068 | 3.35 |
| PM_10_ | O_3_ | -0.01 [-0.03, 0.00] | 0.096 | 3.35 |
| O_3_ | PM_10_ | 0.22 [0.17, 0.26] | <0.01 | 15.05 |
| Executive Function Score | | | | |
| NO_2_ | PM_10_ | -0.29 [-0.37, -0.22] | <0.01 | 13.05 |
| PM_10_ | NO_2_ | -0.02 [-0.05, 0.01] | 0.106 | 3.35 |
| PM_10_ | O_3_ | -0.03 [-0.06, 0.00] | <0.05 | 3.35 |
| O_3_ | PM_10_ | 0.44 [0.36, 0.53] | <0.01 | 15.05 |
